# Ecological relationships of *Haemagogus spegazzinii* (Diptera: Culicidae) in a semiarid area of Brazil

**DOI:** 10.1590/0037-8682-0502-2020

**Published:** 2020-11-25

**Authors:** Cássio Lázaro Silva-Inacio, Anne Aline Pereira de Paiva, Josélio Maria Galvão de Araújo, Maria de Fátima Freire de Melo Ximenes

**Affiliations:** 1 Universidade Federal do Rio Grande do Norte, Programa de Pós-Graduação de Doutorado em Desenvolvimento e Meio Ambiente, Natal, RN, Brasil.; 2 Universidade Federal do Rio Grande do Norte, Laboratório de Pesquisa em Entomologia, Departamento de Microbiologia e Parasitologia, Natal, RN, Brasil.; 3 Universidade Federal do Rio Grande do Norte, Laboratório de Doenças Infecciosas e do Câncer, Departamento de Microbiologia e Parasitologia, Natal, RN, Brasil.

**Keywords:** Haemagogus, Culicidae, Biological cycle, Ecological relationships, Oviposition, Breeding sites

## Abstract

**INTRODUCTION:**

*Haemagogus* are mosquitoes with diurnal habits that live preferentially in forest areas. In Brazil, they are considered the primary vectors of wild yellow fever.

**METHODS:**

The ecological relationships between *Haemagogus spegazzinii*, the environment, and some of its activities in the semiarid region of Rio Grande do Norte were analyzed by collecting eggs with ovitraps, actively searching in tree holes, capturing adults in Shannon traps, and conducting an investigation for viral infections.

**RESULTS:**

A total of 2420 eggs, 271 immature specimens (larvae and pupae), and 206 adults were collected. Egg collection depended on rainfall and relative humidity, with oviposition occurring between January and May. Larvae were found in five plant species, including *Tabebuia aurea* (craibeira)*,* with 160 larvae collected. We observed shared breeding sites between *Hg. spegazzinii* and the following species: *Aedes albopictus*, *Aedes terrens*, *Culex* spp.*,* and *Toxorhynchites theobaldi*. Adults exhibited greater activity between 5 pm and 6 pm, when 191 (92.7%) specimens were captured, while only 1 (0.5%) was collected between 7 pm and 8 pm. The relationship between *Hg. spegazzinii* and rainfall was significant, with positive correlations with accumulated rainfall 5, 10, 15, 20, and 30 days before mosquito collection. We found that the species was infected with the DENV-2 virus.

**CONCLUSIONS:**

This work contributes new information on the bioecology of *Hg. spegazzinii,* with data on the main reproduction periods, oviposition, breeding sites, activity times, and the relationship between the species and meteorological variables in the Caatinga of northeastern Brazil.

## INTRODUCTION

Mosquitoes are widely studied in tropical areas, primarily the Americas, as they harbor viruses, protozoans, and helminths. Among the diseases transmitted by mosquitoes are dengue fever, Zika, chikungunya, yellow fever, and malaria. In tropical environments, such as in Brazil, mosquitoes can occupy different niches and stratifications. Some species of the genera *Haemagogus* and *Sabethes*, which demonstrate a preference for tree canopies, are denominated arboreal^1^. 

In recent years, Brazil has faced arbovirus epidemics. Dengue fever, the most prevalent, had an incidence of 392 cases per 100,000 inhabitants this year of 2020, while, in the same period, chikungunya and Zika had incidences of 22.4 and 2.8 cases per 100,000, respectively^2^. 

At the onset of the Zika epidemic in African and Asian countries, Marcondes and Ximenes^3^ reported on the risk of virus expansion in Brazil, due to high *Aedes* spp. infestation in the country. Zika cases, primarily in the northeast, resulted in serious public health problems caused mainly by microencephaly in newborns^2^.

 Ximenes et al.^4^ recently described the possible involvement of forest mosquito species in a Chikungunya outbreak in Natal, Rio Grande do Norte, where four mosquito species were naturally infected by the Chikungunya virus (CHIKV). 

 Strictly forest dwellers, *Haemagogus*, rarely leave their environment and are primarily active during the day. Most of these mosquitoes inhabit tree holes, bamboo internodes, bromelias, and coconut husks^5^. They lay their eggs on water surfaces and can withstand prolonged unfavorable periods with little rainfall until eclosion. Studies on the desiccation tolerance of *Aedes, Anopheles*, and *Culex* eggs revealed that the darker the egg, the more resistant it is because of the larger amount of melanin and chitin and the serosal cuticle layer^6^ ^,^ ^7^.

Endogenous biological factors and reactions to the external environment have a direct influence on mosquito activities^8^. Thus, light, temperature, humidity, and rainfall are closely related to culicid populations. The climatic differences between seasons and water oxygenation interfere with the life cycle of *Haemagogus*, which may affect the hatching of larvae^8^.

*Haemagogus* are widely distributed throughout the Americas, with 28 known species, some of which transmit yellow fever, Mayaro, and other arboviruses^9^. Nine species have been reported in Brazil, two belonging to the subgenus *Conopostegus*: *Haemagogus leucocelaenus* (Dyar and Shannon, 1924) and *Haemagogus leucophoebus* (Galindo, Carpenter, and Trapido, 1953), and seven to the subgenus *Haemagogus*: *Haemagogus albomaculatus* Theobald, 1903*, Haemagogus baresi* Cerqueira, 1960*, Haemagogus capricornii* Lutz, 1904*, Haemagogus celeste* Dyar and Nuñez Tovar, 1927*, Haemagogus janthinomys* Dyar, 1921*, Haemagogus spegazzinii* Brèthes, 1912, and *Haemagogus tropicalis* Cerqueira and Antunes, 1938. La Corte et al.^10^ found specimens of *Hg. spegazzinii*, and other similar species, in Sergipe state, northeastern Brazil, but the morphology of the male genitalia was different, suggesting the possibility of new species.

Three species were recorded in Rio Grande do Norte, northeastern Brazil: *Hg. leucocelaenus*, *Hg. janthinomys*, and *Hg. spegazzinii* ^11^. The presence of *Hg. janthinomys* raises epidemiological interest, as it is the main species involved in yellow fever transmission in Brazil. The other two species are equally important and have been found naturally infected by the yellow fever virus in other regions and countries^9^. 

Given the importance of this group of culicids, and the lack of information about their distribution on semiarid environments, this study was conducted in the Caatinga (seasonally dry tropical forest) of northeastern Brazil, in order to better understand the biology of *Hg. spegazzinii* based on the ecological relationships of the study site.

## METHODS

### Study area

The collection area was in the municipality of Currais Novos, Rio Grande do Norte (RN) state, 10 km from the city center. Temperatures vary between 18-33 °C, and the average annual rainfall is 610.5 mm. The rainy season is between February and April, sometimes extending to mid-June^12^. The area is known as Cânion dos Apertados (Apertados Canyon) (6°20’31” S 36°30’07” W)^13^, located in the rural area of the municipality, presenting a preserved fragment of Caatinga vegetation (of the Caatinga Hyperxerphile Seridó type).

### Specimen collection

Eggs were collected with ovitraps made in our laboratory between April 2017 and May 2018; 15 traps were placed at five points in a transect, with at least 250 m between them. At each point, 3 traps were positioned vertically at 1 m, 3 m, and 6 m from the ground. A second collection began at the end of this period to observe the egg laying dynamics of this species. The ovitraps were randomly installed in the collection area, at heights varying from 1-3 m. 

**FIGURE 1:**
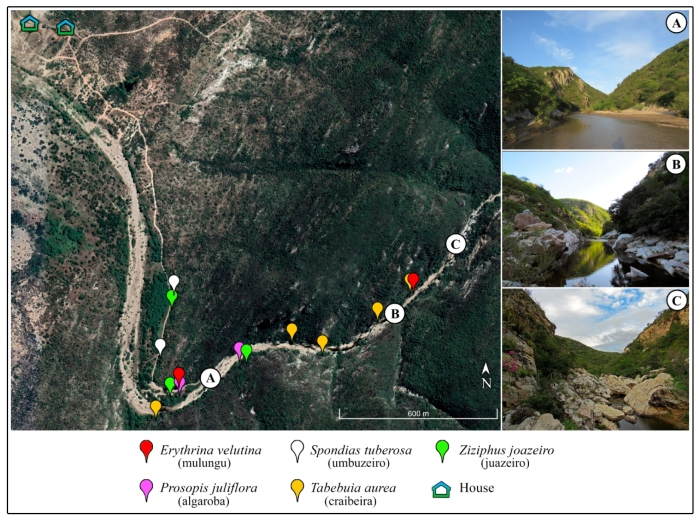
Distribution of plant species used as breeding sites for *Haemagogus spegazzinii* (Brèthes, 1912) in the semiarid region of Rio Grande do Norte state, Brazil. A - C: different landscapes observed in the collection area.

Immature (larvae and pupae) and adult specimens were collected monthly between January 2019 and February 2020. Larvae and pupae were actively collected for three hours (2-5 pm) in tree holes containing water. The insects were captured with a manual suction pump and plastic pipettes, separated into plastic pots, and labeled with the collection site. The water collected was replaced with mineral water. Water volume, height from the ground, and plant species were recorded. The captures were carried out in 14 specimens of 5 tree species: *Erythrina velutina* Willd. (mulungu), *Prosopis juliflora* (Sw.) DC. (algaroba), *Spondias tuberosa* Arruda (umbuzeiro), *Tabebuia aurea* (Silva Manso) Benth. and Hook. f. ex S. Moore (craibeira), and *Ziziphus joazeiro* Mart. (juazeiro) (Figure 1).

Adult mosquitoes were collected between 5-8 pm, in three one-hour intervals, in Shannon traps with a manual capture device.

The law No. 11,794/2008 on the use of animals in research and educational activities deals only with vertebrate animals, and therefore no prior authorization is required for the collection and handling of arthropods. An authorization No. 63521 was obtained for activities for scientific purposes via SISBio. 

### Analysis of the relationship between mosquito abundance and meteorological factors

 Eggs: The Kruskal-Wallis test followed by a Dunn post-test was used to determine the differences between heights in vertical stratification. Pearson’s correlation coefficient was used to establish correlations between mosquito eggs and meteorological variables (wind speed, precipitation, temperature, and relative humidity). A simple linear regression was utilized to explain the influence of meteorological variables on egg abundance. Precipitation 5, 10, 15, 20, and 30 days before collections was analyzed using the Pearson’s correlation coefficient.

Immature specimens: Spearman’s correlation coefficient was used to establish correlations between immature mosquito specimens and meteorological variables (wind speed, precipitation, temperature, and relative humidity) and precipitation 5, 10, 15, 20, and 30 days before collection. 

Adults: Friedman’s test was used to analyze the differences between the times of capture (5-6 pm, 6-7 pm, and 7-8 pm). Pearson’s correlation coefficient was used to establish correlations between adult mosquitoes and meteorological variables (wind speed, precipitation, temperature, and relative humidity). Simple linear regression was utilized to explain the influence of meteorological variables on egg abundance. Precipitation 5, 10, 15, 20, and 30 days before collections was analyzed using the Pearson’s correlation coefficient. 

The meteorological variables (independent variables) were obtained from the National Meteorology Institute (INMET) (Station 82690). Statistical tests were performed using BioEstat v.5.3.

### Biological cycle

In the Entomology Research Laboratory of the Universidade Federal do Rio Grande do Norte (Labent-UFRN), a pallet containing 52 eggs collected in February 2020 was placed in a plastic container and immersed in 2 L of mineral water with 500 mg of dissolved biological yeast (Fermix Dona Benta® - *Saccharomyces cerevisiae meyen* and sorbitan monostearate emulsifier; Macêdo S.A., São José dos Campos, Brazil) for eclosion adapted from Anjolette and Macoris^14^.

Larvae were collected in individual receptacles containing 30 mL of the initial eclosion solution and monitored at the same time daily until reaching the adult stage. The insects were kept at an average temperature of 26 °C and 86% humidity.

### Determination of viral infections

Adult mosquitoes collected in the Shannon trap were identified, separated into samples, and subjected to viral extraction using the QIAmp Viral MiniKit (QIAGEN, Inc., Valencia, CA, USA), according to the manufacturer’s protocol. The cDNA was amplified by RT-Nested-PCR for flavivirus detection, as previously described^15^. Sequencing was conducted using the BigDye Terminator Cycle Sequencing Ready Reaction kit (Applied Biosystems, Beverly, MA, USA) and analyzed using an ABI Prism 3730 Sequencer (Applied Biosystems). The electropherograms were visualized using Chromas version 1.45 (Technelysium Pty. Ltd., Queensland, Australia), and the nucleotide sequences were submitted to Nucleotide Basic Local Alignment Search Tool (BLAST)^16^ for viral identification.

## RESULTS

### Eggs

The first egg collection period (April 2017 to May 2018) revealed that the eggs were laid at heights of 1 m and 3 m. A total of 193 eggs were collected. There was no statistically significant difference between egg collection heights (H = 1.7335, *p* = 0.4203). Nevertheless, we decided to install the traps between 1-3 m above the ground. The Spearman’s correlation coefficient did not show a relationship between meteorological variables and mosquito egg abundance for this period (*p >*0.05). 

**FIGURE 2:**
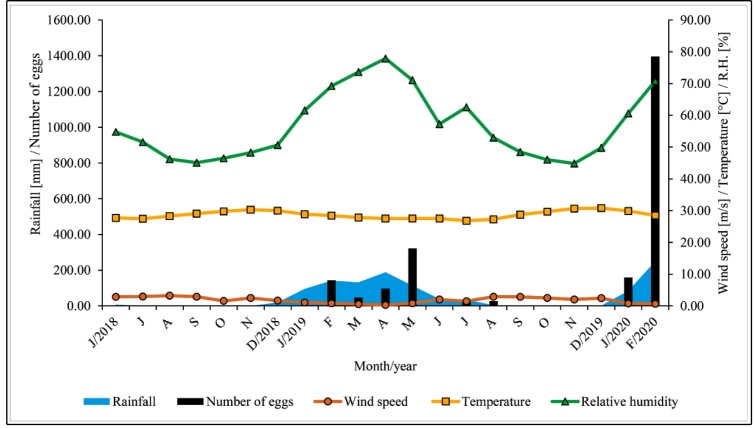
Relationship between meteorological variables (rainfall, wind speed, temperature, and relative humidity) and *Haemagogus spegazzinii* oviposition, between June 2018 and February 2020, in the Brazilian semiarid region.

In the second period (June 2018 to February 2020), 2227 eggs were collected (Figure 2). The influence between meteorological variables and egg abundance was analyzed from April 2017 to February 2020. Pearson’s correlation showed significant results with the meteorological variables of wind speed (r = -0.354*, p* = 0.0368) and rainfall (r = 0.4479, *p =* 0.0069), but no significant correlation with temperature (r *=* -0.0646*, p =* 0.7124) or relative humidity (r = 0.3264*, p* = 0.0556). Simple linear regression, which seeks to explain which variables influence mosquito egg abundance, showed that the number of eggs was affected by wind speed (Coeff. (b) = -81.04, *p =* 0.0368) and rainfall (Coeff. (b) = 1.2528, *p =* 0.0069). The estimated regression showed that for each additional meter per second in wind speed, there was an average decrease of 81.04 eggs. Regarding precipitation, the estimated regression revealed that, on average, there was an increase of 1.25 eggs for each millimeter of additional rain. Peak oviposition occurred between January and May. 

The relationship with precipitation before collection days was positively correlated with periods of rain of 5 (r = 0.7165, *p* <0.0001), 10 (r = 0.7303, *p* <0.0001), 15 (r = 0.6122, *p* <0.0001), 20 (r = 0.5902, *p* = 0.0002), and 30 (r = 0.5128, *p* = 0.0016) days.

### Immature specimens

**FIGURE 3:**
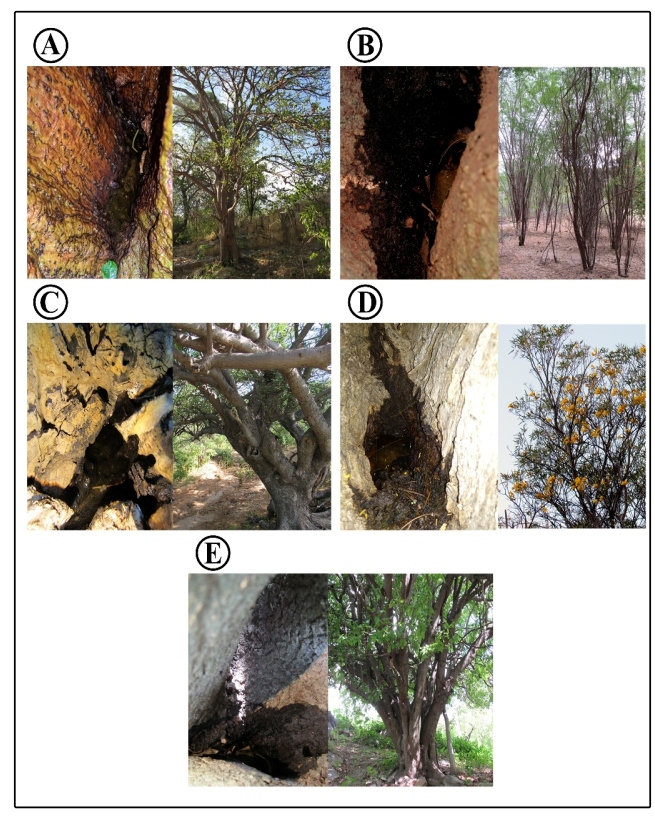
Mosquito breeding sites in tree holes in the semiarid region of northeastern Brazil. **A:** *E. velutina* (mulungu); **B:** *P. juliflora* (algaroba); **C:** *S. tuberosa* (umbuzeiro); **D:** *T. aurea* (craibeira); **E:** *Z. joazeiro* (juazeiro). Panels show close-up pictures of the breeding sites (left) and pictures of the whole trees (right).

A total of 261 immature *Hg. spegazzinii* specimens were collected between January 2019 and February 2020 in five Caatinga tree species: *E. velutina*, *P. juliflora*, *S. tuberosa*, *T. aurea*, and *Z. joazeiro*. (Figure 3). The highest volume of water in the breeding sites was observed in *T. aurea* trees (341 mL), followed by *S. tuberosa* (83 mL), and the lowest volume in *E. velutina* (36 mL). The highest breeding site above the ground was *S. tuberosa* (163 cm), and the lowest *Z. joazeiro* (32 cm). *Hg. spegazzinii* sharing of breeding sites was observed in *T. aurea*, with *Aedes terrens* (Walker, 1856)*, Toxorhynchites theobaldi* (Dyar and Knab, 1906)*,* and *Culex* spp.; and in *P. juliflora*, with immature *Aedes albopictus* (Skuse, 1895) (Table 1). 

**TABLE 1:** Plant species used as breeding sites by *Haemagogus spegazzinii*, in the semiarid region of Rio Grande do Norte, Brazil, and other mosquito species that share the same breeding sites.

Plant species	Common	Immature	Height from ground	Maximum volume	Other
	name	*Hg. spegazzinii*	level (cm)	(mL)	species
*Erythrina velutina* Willd.	Mulungu	18	124	36	-
*Prosopis juliflora* (Sw.) DC.	Algaroba	56	38	70	*A. albopictus*
*Spondias tuberosa* Arruda	Umbuzeiro	14	163	83	-
*Tabebuia aurea* (Silva	Craibeira	160	157	341	*A. terrens*
Manso) Benth. and Hook.					*Culex sp.*
f. ex S. Moore					*T. theobaldi*
*Ziziphus joazeiro* Mart.	Juazeiro	13	32	52	-

Spearman’s correlation showed that wind speed was inversely proportional to the abundance of immature individuals (rs = -0.8131, *p* = 0.0004). The other meteorological variables exhibited a significant positive association with the abundance of immature mosquitoes, precipitation (rs = 0.7199, *p* = 0.0037), and relative humidity (rs = 0.7191, *p* = 0.0037). Temperature did not exhibit a statistically significant impact (rs = -0.188, *p* = 0.5198). 

Precipitation for 5 (rs = 0.6703, *p* = 0.0087), 15 (rs = 0.6208, *p* = 0.0178), 20 (rs = 0.7199, *p* = 0.0037), and 30 (rs = 0.741, *p* = 0.0024) days prior to collection was positively associated with abundance of immature specimens. 

### Adults

**FIGURE 4:**
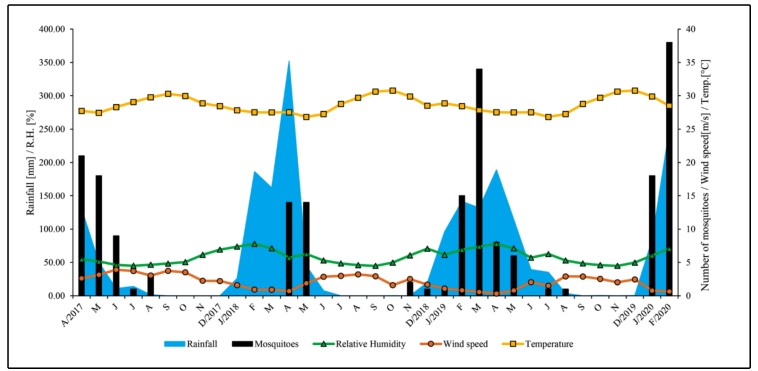
Monthly distribution of adult *Haemagogus spegazzinii* mosquitoes in the Brazilian semiarid regions and its relationship with meteorological variables (rainfall, relative humidity, wind speed, and temperature).

Between April 2017 and March 2020, we collected 206 mosquitoes (all females), 191 (92.7%) of which were captured between 5-6 pm; 14 (6.8%) between 6-7 pm and 1 (0.5%) between 7-8 pm. The Friedman’s test revealed significant differences between the collection times (Fr = 12.1, *p* = 0.0024), 5-6 pm and 6-7 pm (*p* <0.05), and 5-6 pm versus 7-8 pm (*p* <0.05). However, no significant difference was observed between 6-7 pm and 7-8 pm (*p* >0.05). The number of mosquitoes and meteorological variables analyzed by Pearson’s correlation demonstrated a relationship between three of the four variables investigated. Wind speed had a negative influence on the number of mosquitoes; that is, the higher the wind speed, the lower the number of mosquitos captured (r = -0.4093, *p* = 0.0146). Since rainfall showed a positive correlation with the number of mosquitoes, the higher the rainfall, the more mosquitoes were captured (r = 0.5919, *p* = 0.0002) (Figure 4).

Simple linear regression seeks to explain which independent variables affect the local mosquito population (dependent variable). Analysis revealed a relationship between meteorological variables and mosquito abundance (wind speed: Coeff. (b) = -3.8474, *p =* 0.0146; precipitation: Coeff. (b) = 0.068, *p* <0.0001). Therefore, it is estimated that, on average, for each additional meter per second in wind speed, there is a decrease of 3.8 mosquitoes, and for each additional millimeter of rain, there is an increase of 0.068 mosquitoes. 

Rainfall has the strongest correlation with the increase in species abundance. Analyses of accumulated rainfall in the days preceding collection revealed a correlation with all the periods analyzed (5 days: r = 0.4302, *p* = 0.0098; 10 days: r = 0.4402, *p* = 0.0081; 15 days: r = 0.4454, *p* = 0.0073; 20 days: r = 0.497, *p* = 0.0024; 30 days: r = 0.555, *p* = 0.0005). 

For all regressions performed, the residuals satisfied the assumptions about the errors of the linear regression model.

### Biological cycle

The mosquitoes completed their life cycle, from larval hatching to the adult stage, in 14 days. The first larvae were observed 24 h after egg immersion, took up to 5 days to reach the fourth instar (L4), and up to 12 days to become pupae. The first adults to emerge were male, 12 days after immersion, while females appeared only after 14 days. All the insects developed completely, resulting in 20 males and 17 females.

### Viral infections

A viral analysis was conducted in 136 female *Hg. spegazzinii* mosquitoes, separated into 20 samples. Only one sample, collected in June 2017, containing 7 mosquitoes, was positive for the dengue fever virus (DENV-2). The other samples were not infected with flaviviruses.

## DISCUSSION

*Haemagogus* mosquitoes, widely distributed in Brazil^9^, are a well-studied group in the Atlantic and Amazon Forests^17^ ^-^ ^21^. However, in the Brazilian semiarid region, where vegetation and climate conditions are very different from those in the rest of the country, no specific studies have been conducted on this epidemiologically important group in the context of yellow fever.

Four species are known to inhabit the northeast of Brazil: *Hg. leucocelaenus, Hg. spegazzinii, Hg. janthinomys*, and *Hg. capricornii* ^5^ ^-^ ^9^. However, only the first three have been recorded in the Rio Grande do Norte state^11^. 

The biology of *Haemagogus* species seems to overlap and is always reported as being similar for the entire genus^5^. However, regional variations between populations have not been considered, which leaves a gap in the understanding of the biology of these mosquitoes.

*Hg. spegazzinii* is widely distributed in South America, including in Argentina, eastern Bolivia, and northeastern and southeastern Brazil. Although it is not recognized as a primary vector of yellow fever in Brazil, this species coexists with *Hg. janthinomys* and has been found to be naturally infected by the yellow fever virus^9^. A study conducted in Maranhão state, during a yellow fever outbreak in 1993 and 1994, found the natural infection of four species, namely, *Hg. janthinomys*, *Hg. albomaculatus* Theobald, 1903*, Sabethes chloropterus* (von Humboldt, 1819), and *Sabethes soperi* Lane & Cerqueira, 1942. The first of which had the highest infection percentages^22^ *.* Samples of *Hg. spegazzinii*, collected in the present study, were submitted for viral investigation, and DENV-2 was detected for the first time in this species. This result raises important questions about the possible persistence of the dengue virus in forest-dwelling mosquitoes^23^. It has been reported that the dengue fever virus may adapt to *Haemagogus*, thereby increasing the interest in studies on forest mosquitoes^9^. For example, some studies have indicated the ability of *Hg. leucocelaenus* to be infected with and transmit the CHIKV virus, and other species can infect and transmit the virus causing Mayaro fever^21^ ^,^ ^24^.

The habitat diversity of immature individuals observed for this species likely reveals its adaptation to the semiarid region of northeastern Brazil. A recent study broadened the geographic distribution of *Hg. spegazzinii,* extending to the province of La Pampa in Argentina, where a local breeding site of the species was observed in a hole of *Prosopis caldenia* Buckart (calden) just above ground level, revealing the most southern distribution of the genus *Haemagogus* ^25^. 

In the present study, we found that peak egg-laying is related to the occurrence adult peaks. This can be explained by the greater female activity during the rainy season. Eggs were always laid in this period and peak collections were related to the rainfall that occurred 5-30 days before. Abundance of adults also exhibited stronger correlations with rainfall 20-30 days before collection. 

Under laboratory conditions, 20 *Hg. leucocelaenus* larvae took an average of 10.44 ± 0.70 days to reach the adult stage, at a temperature of 28 ± 1 °C and relative humidity between and 75-90%^26^. In the present study, we observed field temperatures varying between 26.8-30.7 °C, and relative humidity between 44.8-77.9%. These conditions may accelerate larval eclosion and development, resulting in rapid adult emergence. At laboratory temperatures between 24.1-27.5 °C, and humidity ranging between 76-97%, the 37 larvae took an average of 12.5 days to develop to the adult phase in our study. This difference of approximately 2 days may be due to the temperature variation during our observations, with the insects being housed at ambient temperature.

 Rainfall, temperature*,* and relative humidity influence the life cycle of mosquitoes, including *Hg. leucocelaenus* and *Hg. janthinomys* ^5^ ^,^ ^26^ ^-^ ^30^. The same was observed for *Hg. spegazzinii* in the present study. Under favorable environmental conditions, mosquito populations develop rapidly. On the other hand, the lack of food and high larval density may prolong this phase, as observed in *Aedes aegypti,* influencing the density of future populations^31^ ^,^ ^32^.

Natural receptacles are the main breeding sites of *Haemagogus* species, such as tree hollows, cut bamboo, coconut husks, and bromelias^9^. In the present study, we identified five plant species typical of the Caatinga, a seasonally dry tropical forest, as development sites. *Hg. spegazzinii* is apparently a generalist in terms of the plant species it colonizes and frequently dominates. In the semiarid region, where trees are small, these mosquitoes tend to lay their eggs in breeding sites with a small opening, located in shady areas up to 3 m from the ground, in order to reduce evaporation.

The species also lays its eggs in artificial receptables, such as the plastic pots used in the ovitraps of this study and an earlier investigation^33^, perhaps resulting from a survival strategy in a dry environment. This differs from the arboreal behavior reported by authors in other regions of the country^5^ ^,^ ^9^ ^,^ ^34^. *Haemagogus* oviposition occurred mainly at temperatures that varied between 27.5-29.9 °C, with relative humidity between 60.5-77.8% during the rainy season. That is, with environmental conditions that favor the development of the insect. In an area of the Atlantic Forest, in Rio Grande do Norte, the oviposition of *Hg*. *leucocelaenus* was observed to start at the first rainfall peak of the year and occur near the ground, even in high vegetation^27^. 

The main breeding sites were found in *T. aurea*, a thick-barked tree with height varying between 5-20 m and diameter of up to 1 m. Present across almost the entire country^35^, in the northeast, it occurs primarily in areas of the ciliary forest and limestone-saline soils. The plant species was also colonized by *Haemagogus* sp. nr. *spegazzinii* Brèthes, 1912, *Aedes terrens* (Walker, 1856), *Culex conservator* Dyar and Knab, 1906, and *Toxorhynchites* spp. in the Caatinga of Sergipe state, Brazil^10^. In the present study, the highest number of breeding sites (5) and the largest amount of accumulated water (341 mL) were found in this tree.

Mosquitoes generally obtain their nutrients from plants, and only females are hematophagous. Some *Haemagogus* species, such as *Hg. capricornii, Hg. leucocelaenus*, and *Hg. janthinomys*, have blood meals from different animals, feeding on birds, rodents, cattle, non-human primates, and humans^9^. *Hg. spegazzinii* captures occurred mainly in the first time interval (5-6 pm), reinforcing the diurnal activity of the genus^5^ ^,^ ^9^. However, diurnal or nocturnal circadian rhythms can only be confirmed with successive 24-hour captures.

 Observations on the development of *Hg. spegazzinii* may explain the population dynamics of the species during the year. Immature individuals need 8 to 14 days to become adults. 

The rainfall recorded 15, 20, and 30 days before collection influenced mosquito development. The primary emergence of male mosquitoes likely results from the time required for reproductive organ development and subsequent copulation^5^. 

The present study adds information on the bioecology of *Hg. spegazzinii*, with data on the main reproduction periods, egg-laying stratum, breeding sites, and activity times of the species in the Brazilian semiarid region as well as the relationship between the species and the local meteorological variables. With respect to the breeding sites for *Hg. spegazzinii,* the first record of the species colonizing seasonally dry tropical forest trees were reported for northeastern Brazil.

Studies that broaden the knowledge about the possibility of this mosquito species participating in the transmission and persistence of flavivirus, considering the differences between the country’s biomes and the particularities of the species and populations, are important, since *Hg. spegazzinii* is widely distributed throughout Brazil.
